# Auditory mechanics in the grig (*Cyphoderris monstrosa*): tympanal travelling waves and frequency discrimination as a precursor to inner ear tonotopy

**DOI:** 10.1098/rspb.2022.0398

**Published:** 2022-04-27

**Authors:** Charlie Woodrow, Christian Pulver, Hojun Song, Fernando Montealegre-Z

**Affiliations:** ^1^ School of Life Sciences, Joseph Banks Laboratories, University of Lincoln, Green Lane, Lincoln LN6 7DL, UK; ^2^ Department of Entomology, Texas A&M University, 2475 TAMU, 77843-2475 College Station, TX, USA

**Keywords:** hearing, tonotopic, ear, Orthoptera, haglid, relict

## Abstract

Ensiferan orthopterans offer a key study system for acoustic communication and the process of insect hearing. *Cyphoderris monstrosa* (Hagloidea) belongs to a relict ensiferan family and is often used for evolutionary comparisons between bushcrickets (Tettigoniidae) and their ancestors. Understanding how this species processes sound is therefore vital to reconstructing the evolutionary history of ensiferan hearing. Previous investigations have found a mismatch in the ear of this species, whereby neurophysiological and tympanal tuning does not match the conspecific communication frequency. However, the role of the whole tympanum in signal reception remains unknown. Using laser Doppler vibrometry, we show that the tympana are tonotopic, with higher frequencies being received more distally. The tympana use two key modalities to mechanically separate sounds into two auditory receptor populations. Frequencies below approximately 8 kHz generate a basic resonant mode in the proximal end of the tympanum, whereas frequencies above approximately 8 kHz generate travelling waves in the distal region. Micro-CT imaging of the ear and the presented data suggest that this tonotopy of the tympana drive the tonotopic mechanotransduction of the *crista acustica* (CA). This mechanism represents a functional intermediate between simple tuned tympana and the complex tonotopy of the bushcricket CA.

## 1. Introduction

The success of a communication system depends on the coevolution of participants of two transient categories—the signallers and the receivers [1,2]. In complex signal rich environments, picking out relevant signals from the surrounding noise is no trivial task, requiring multiple elements of discrimination or tuning at different stages of the signal reception process. In this way, we could describe the mechanical elements of a receiver as possessing several degrees of freedom, each of which contribute individually, or together, to aid in successful perception of relevant signals.

In the case of acoustic communication, interpreting the various signals of the surrounding environment requires the ability to discriminate between sounds of differing temporal and frequency compositions, as this information will allow the receiver to discern whether the signaller is a conspecific, prey opportunity, or threat, and thus how to respond behaviourally to maximize fitness and survival [2,3]. Though deciphering the temporal information of an acoustic signal requires some level of central processing, the separation of useful and unnecessary frequency information can be easily enhanced by biophysical tuning of the mechanical components of the ear.

In the case of many vertebrates, frequency discrimination is achieved by tonotopy of the inner ear, whereby the mechanical transduction of sound is frequency and place dependent. The best-known example is the mammalian cochlea, which separates acoustic signals of differing frequencies into appropriate receptors by means of mechanical and physiological tuning [4,5]. Tonotopic sound receptor organs are also observed in amphibians, reptiles and birds [6,7]. However, auditory frequency discrimination is not unique to vertebrates. Many insects also possess complex receivers for frequency processing, either mechanically on the tympanum [8,9] or at the mechanosensory units [10,11]. On the other hand, many insects lack ears, or have simple ears with a reduced ability to discriminate between sound frequencies. In the receivers of the latter, we tend to observe extremely efficient tuning of the whole system to discrete signal channels [12,13]. Insect ears thus provide a model system for investigating the mechanical differences between complex frequency discrimination systems and simple tuned receivers, which could in turn allude to the global mechanisms that govern the evolution of tonotopic receiver organs.

In the Ensifera (bushcrickets, crickets and allies), we observe both instances of simple biophysical tuning, and complex frequency discrimination. Their ears are found on the proximal ends of the foretibiae, and in bushcrickets (Tettigoniidae) consist of two paired tympana, backed by two air-filled tracheal branches, which can receive sound externally directly on the tympana, or via a narrowing ear canal originating from the prothoracic spiracle [14–17]. The tympana act as pressure-difference receivers, converting airborne sound from multiple inputs into a single mechanical travelling wave in the inner ear, or *crista acustica* (CA), which lies above the anterior tracheal branch. The bushcricket CA has long been regarded as an analogous system to the mammalian cochlea [10,11,18–20], separating incoming acoustic signals by their frequency information. However, in many ensiferans, acoustic communication among conspecifics is simply facilitated by a parallel between the frequency composition of the conspecific acoustic signals and the frequency response of the ear. This coupling has been exemplified both at the mechanical tuning of tympanic membranes [21,22], and in the relative tuning and sensitivity of primary auditory receptors [19,23,24]. In some species, however, mismatches between signaller and receiver occur [25–27]. This is believed to be the case in the relict ensiferan *Cyphoderris monstrosa* (Ensifera: Hagloidea). In this species, the male song, produced by tegminal stridulation, consists of a series of pure tone trills at a carrier frequency of 11–13 kHz, while the mechanical and neurological elements of the ear have been found to have their lowest sensitivity threshold at approximately 2 kHz [26,28]. Recordings of the primary auditory receptors in this species have also revealed the presence of two functional receptor types. Some are tuned to low frequencies (up to about 8 kHz), while others are tuned more broadly (up to about 20 kHz [26]). It is believed that this ear therefore possesses mismatched tuning but is capable of a simple level of frequency discrimination. Proposed explanations for the mismatch between neuronal tuning and conspecific communication focused on the precursory role of the foretibial organ in the detection of substrate-borne vibrations [28,29]; however, the extent of tuning across the whole tympanum has previously not been considered in such a hypothesis. In addition, the mechanics of hearing across the Ensifera remains unexplored from a comparative perspective yet understanding the variation in auditory mechanics between species could allude to the evolution of their complex hearing systems.

As *C. monstrosa* is one of only eight extant species in the family Prophalangopsidae, a group dominant during the Jurassic [30,31] with over 100 fossil species [32], it is often used for evolutionary comparisons between ancient and modern ensiferans. A recent phylogenomic study of Orthoptera recovered a robust sister relationship between Prophalangopsidae and Tettigoniidae, which diverged in the Jurassic [33]. Therefore, a critical comparison of the hearing systems between the two families will reveal important insights into the evolution of acoustic communication and hearing. Here, we set out to re-visit the peripheral hearing system of *C. monstrosa* to revise hypotheses regarding its mismatch. Using micro-CT imaging and laser Doppler vibrometry, we re-describe the mechanical features of the ear of *C. monstrosa*, providing biophysical evidence that the tympana are tonotopically arranged, and key to frequency discrimination. This mechanism offers insights into a crucial step in the evolution of the complex ear of the tettigoniid.

## 2. Methods

### (a) Specimens

*Cyphoderris monstrosa* (eight males, one female) were hand-captured in the pine forest near Grayback Gulch Campground, Boise, ID, USA (43°48'25.4″ N, 115°51'59.9″ W), on 5 June 2021 by H.S. and sent to the University of Lincoln, UK, for characterization. A further set of specimens (three males, six females) were collected in William A. Switzer Provincial Park, Alberta, Canada (53°29'0.51″ N, 117°49'32.55″ W) between 6 and 13 July 2019 by an external collaborator, as part of a project on temporal and geographic variation. A subset of these were sent to the University of Lincoln, UK, for this study.

While at the University of Lincoln, all specimens were maintained on an ad libitum diet of bee pollen (Sevenhills, Wakefield, West Yorkshire, UK), fresh carrot, and cat biscuits (James wellbeloved, Somerset, UK) and had access to water. Each animal was kept in an individual container in a cooled 24 h incubator (PHCbi MIR-154, PHC Holdings Corporation, Tokyo, Japan) on a four-step temperature cycle (00.00, 8°C; 06.00, 10°C; 12.00, 15°C; 18.00, 10°C) and a 10 h : 14 h light/dark cycle.

### (b) µ-CT imaging

Most specimens were maintained in the colony until natural death by senescence for use in other studies, but for micro-CT imaging, one male and one female were euthanized by placement in 90% ethanol. These specimens were scanned using a SkyScan 1172 μ-CT scanner (Bruker Corporation, Billerica, MA, USA). The forelegs were removed and mounted in custom built holders before scanning (voxel size 2 µm, 55 kV, 180 µA, 800 ms exposure, 0.1° rotation step). μ-CT projection images were reconstructed to produce orthogonal slices with NRecon (v. 1.6.9.18, Bruker Corporation, Billerica, MA, USA). For three-dimensional segmentation of the trachea, the slice data were imported into Amira-Aviso 6.7 (Thermo Fisher Scientific, Waltham, MA, USA) and the trachea manually selected using the magic wand tool every five slices throughout the whole foreleg, followed by interpolation to connect the selected geometries and generate a three-dimensional surface.

### (c) Laser Doppler vibrometry

The frequency response of the tympana was measured in seven female and 11 male specimens of *C. monstrosa,* totalling 36 individual ears of which 33 provided suitable data for analysis. Specimens were immobilized using a unique method under previous protocols [34]; by freezing at −2°C for 2 min. They were then mounted in a natural orientation to a copper platform with wax made of 50% beeswax (Fisher Scientific, Loughborough, UK) and 50% colophonium (Sigma-Aldrich Company Ltd, Dorset, UK). Specimens were allowed time to recover from the immobilization method prior to data collection.

Tympana responses were measured using a micro-scanning LDV system (PSV-500, Polytec GmbH, Waldronn, Germany), with approximately 600 grid points at a sampling frequency of 512 kHz. Broadband periodic chirps of 2–60 kHz were generated within Polytec laser software (Polytec GmbH, Waldbronn, Germany). These signals were amplified (A-400, Pioneer, Kawasaki, Japan) and transmitted to a RAAL 140–15D Flatfoil loudspeaker (RAAL advanced loudspeakers, Zaječar, Serbia and Montenegro) positioned 20 cm from the specimen, ipsilateral to the ear being scanned. The amplitude of each stimulus was corrected within the software to deliver a flat frequency response. The broadband stimulus was delivered at 60 dB SPL, as this provided the best frequency response of the speaker. While neural thresholds at high frequencies in this system can exceed 60 dB SPL, the tympanum response in bushcrickets, most gryllids, and this species, is linear [28,35], thus we did not have concerns about the SPL for a purely biomechanical investigation. For pure tone experiments, four-cycle pure tone stimuli at either 12.5 kHz or 2 kHz were generated with a waveform generator (SDG 1020, Siglent, China). A 2 ms signals containing the four-cycle tone were averaged 20 times during acquisition for each of the approximately 600 scan points. A reference signal for stimulus calibration and gain calculation was recorded using a B&K 1/8″ Type 4138 omnidirectional microphone (Brüel & Kjær, Nærum, Denmark) positioned 1 cm dorsal of the foretibial join. Data were acquired within the Polytec 9.4 acquisition software and saved in its original format as well as.txt format for analysis. For all distance-based measurements, the data were normalized prior to analysis to account for minor variations in tympanum size.

### (d) Data analysis and characterization of travelling waves

To statistically confirm the effect of stimulus frequency on tympanum velocity and location of maximum displacement, a two-way ANOVA model was computed using the lmerTest package in R v. 4.1.0 [36,37]. Stimulation frequency and relative distance along the tympanum (normalized to control for individual tympanum size) were considered fixed factors, and their interaction was included to differentiate between additive and interactive effects on velocity. This test was chosen because the frequency variable was considered a categorical factor and the two fixed factors were not normally distributed. Calculations of travelling wave velocity and wavelength followed existing protocols [9,38] and can be found in more detail in the electronic supplementary material. The effect of frequency on travelling wave velocity and wavelength was also statistically assessed by means of a linear ANOVA model using the aforementioned R package.

## 3. Results

### (a) Morphology of the foretibial organ

**Figure 1.**
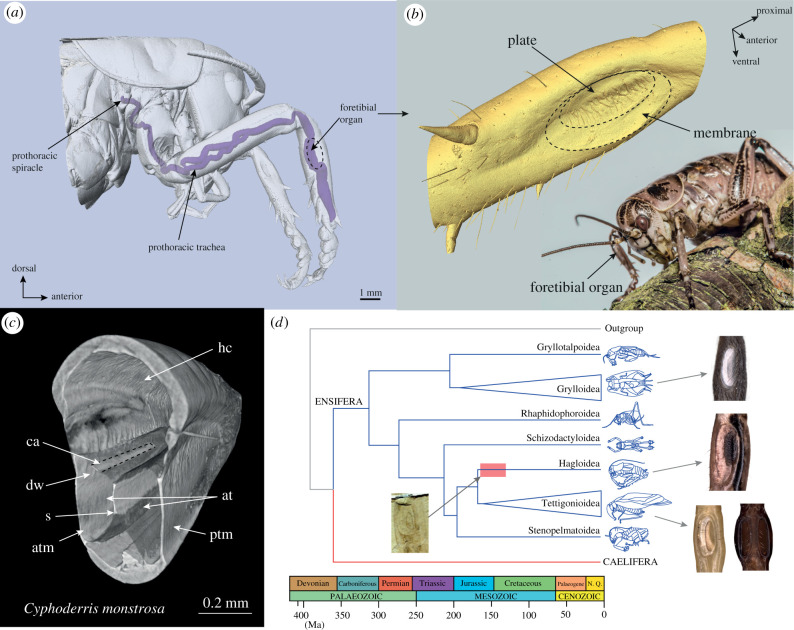
Morphology of the foretibial organ of *Cyphoderris monstrosa* and phylogenetic context. (*a*) Location of the foretibial organ and anatomy of the prothoracic trachea, which is not specialized for sound conduction in this species. (*b*) External anatomy of the foretibial organ, with the two key parts of the tympanum highlighted. (*c*) Cross-sectional anatomy of the foretibial organ of *C. monstrosa*. (*d*) Phylogeny of tympanal ears in the Ensifera mentioned in this study, with an example of a Jurassic prophalangopsid (Hagloidea) indicated with arrow. Labels: ca, *crista acustica*; dw, dorsal wall; atm, anterior tympanal membrane; ptm, posterior tympanal membrane; s, septum; hc, haemolymph channel (liquid filled); at, acoustic tracheae (air-filled). *d* modified from Song *et al.* [33]. Fossil prophalangopsid in *d* from Gu *et al.* [31]. (Online version in colour.)

Like the tettigoniids, *C. monstrosa* possesses two paired tympana (anterior and posterior, or ATM and PTM, respectively) on each foreleg, backed by air-filled trachea, at the proximal ends of the foretibiae (figure 1*a*,*b*). The tympana have a thick region, and a thinner membranous region along the ventral edge. *Cyphoderris monstrosa* possesses a valved prothoracic spiracle which, in most tettigoniids, has become open and much larger [14]. It has been suggested that in gryllids, the opening/closing of valved spiracles is not related to an auditory function [39]. In *C. monstrosa*, valved spiracle is the opening to three respiratory tracheal branches including an unspecialized prothoracic branch (figure 1*a*) which in the tettigoniids forms an exponential horn to deliver sound internally to the tympana [14–16,40]. Sound reception in *C. monstrosa* is not specialized through this trachea but only externally on the tympanum surface [26]. Internally, the ear consists of two enlarged branches of the tracheal system with the auditory chordotonal organ, the CA, lying dorsally above the anterior tracheal branch (figure 1*c*). In *C. monstrosa*, these branches are symmetrical. In the Tettigoniidae, there is an asymmetry that favours the anterior branch, which displays a flat and unilaterally widening dorsal surface, referred to as the dorsal wall (DW). Such a morphological specialization of the DW is not present in *C. monstrosa* (figure 1*c*). Externally, the foretibial organ resembles those of the Jurassic Prophalangopsidae more than those of extant relatives (figure 1*d*).

### (b) Tympanum tonotopy and responses to broadband stimulation

**Figure 2.**
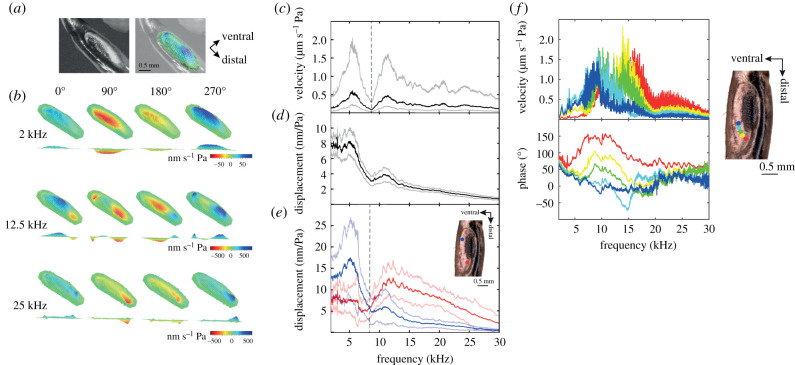
Micromechanics of the anterior tympanum (ATM) of *C. monstrosa*. (*a*) Image of the tympanal organ as seen through the LDV software, showing the orientation of the displacement map. (*b*) Velocity of the tympanum at different frequencies, through one phase cycle. (*c*) Averaged velocity response across all scan points of the tympana (*n* = 33). (*d*) Averaged displacement response across all scan points of the tympana (*n* = 33). (*e*) Averaged displacement response at proximal and distal locations of the tympana. (*f*) Velocity and phase responses of one individual in the high frequency distal region of the tympanum, demonstrating the tonotopy of the frequency response and the increasing phase lag from distal to proximal characteristic of a travelling wave. Dark lines in (*c–e*) indicate mean, and lighter lines indicate ± s.d. Dotted lines on (*c*) and (*e*) demonstrate the average frequency at which the displacement mode changes to become a travelling wave. (Online version in colour.)

Using broadband periodic chirps ranging from 2 to 60 kHz, the frequency response of the tympana was identified (figure 2). The tympana of *C. monstrosa* are responsive to frequencies ranging from approximately 2 to approximately 30 kHz (figure 2*c–e*). Unlike previous findings, we present evidence that the tympana display unique modalities that differ depending on the stimulus frequency. In response to sound frequencies below 8.12 ± 0.47 kHz (*n* = 33 ears), the maximum displacement of the tympanum is towards the middle-proximal end of the ear with a basic vibrational mode (figure 2*b*). However, when the frequency of stimulation is above this value, the modality of the tympanum changes to become a travelling wave propagating from the distal end of the tympanum to the middle of the tympanum (figure 2*b*). These two mechanical modes are also represented in the frequency responses of the tympana, with the average velocity dropping at around 8 kHz (figure 2*c*). The difference is less pronounced in the average displacement response of all scan points (figure 2*d*) but is very clear when the region of measurement is considered in the displacement response (figure 2*e*). In the distal end of the tympanum, at frequencies above 8.12 ± 0.47 kHz (*n* = 33 ears), the response also appears to be tonotopic, with higher frequencies showing a maximum displacement towards the distal end of the ear (figure 2*f*). The travelling waves dissipate at frequencies above 25 kHz, where the reduced response suggests the tympana do not function for the efficient reception of sounds above this frequency (figure 2*c–f*). The phase response of the tympanum also displays an increasing lag towards the proximal end (figure 2*f*), indicative of a travelling wave. In the high frequency region of the tympana, the maximum tympanum displacement is at a frequency of 12.55 kHz (figure 2*e*, *n* = 33 ears), which matches the range of the calling song of this species. In the low frequency region, the maximum tympanum displacement is at 5.25 kHz (figure 2*e*, *n* = 33 ears). We did not observe any unique displacement of the thicker region of the tympanum.

### (c) Tympanum response to pure tone stimuli

**Figure 3.**
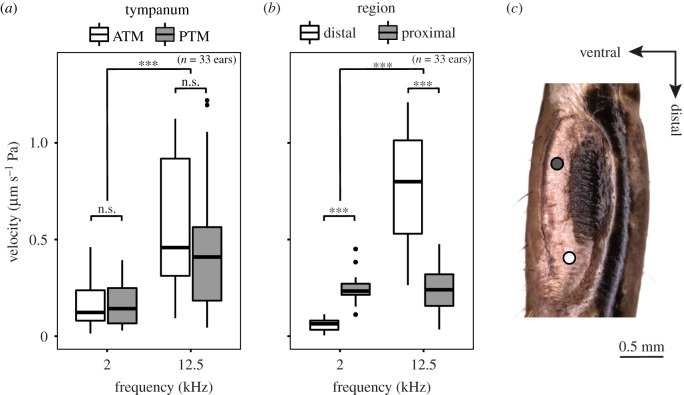
Responses to pure tones in the tympana of *C. monstrosa*. (*a*) The effect of stimulus frequency on velocity by tympanum. (*b*) The effect of stimulus frequency on velocity by region measured. (*c*) Region of the tympanum measured for proximal and distal comparisons. ATM, anterior tympanic membrane; PTM, posterior tympanic membrane; *** = *p* < 0.001; n.s., not significant. (Online version in colour.)

As well as stimulating the system with broadband stimuli, four-cycle pure tone stimuli were presented to the ear and recorded in the time domain to ensure the tonotopy was not an artefact of the broadband stimulation. Owing to the previous works on this species [26,28], the frequencies chosen for pure tone stimulation were 12.5 kHz (the average peak calling song frequency of the species under our rearing conditions; electronic supplementary material, figure S1) and 2 kHz (which has been previously found to be the best tuning of the auditory sensory neurons). Pure tone responses confirmed the observation seen in the broadband stimulation experiments (figure 2), whereby the maximum region of displacement of the tympanum differed depending on the frequency of stimulation, with the 2 kHz tone displaying maximum velocity of 151.9 ± 105.6 nm s^−1^ Pa in the proximal end of the tympanum, and the 12.5 kHz tone displaying a maximum velocity of 508.1 ± 339.7 nm s^−1^ Pa in the distal region (figure 3*a*). At each of these frequencies, there was no evidence supporting differences in tympanum velocity between the ATM and PTM (figure 3*a*; ANOVA, *f* = 0.86, *p* = 0.356), but very strong evidence that tympanum vibration velocity is dependent on the region of the tympanum measured (figure 3*b*; ANOVA, *f* = 36.5, *p* < 0.001).

### (d) Characterization of travelling waves

**Figure 4.**
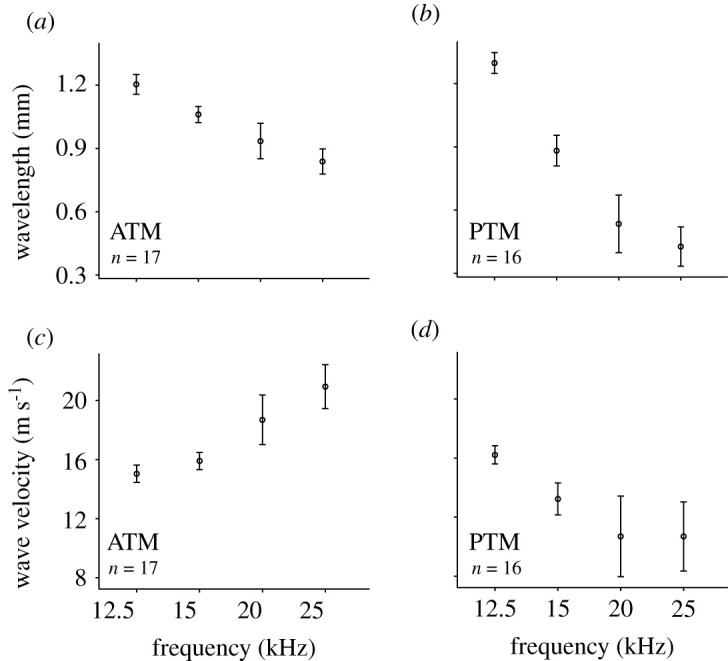
Description of high frequency travelling waves in the tympana of *C. monstrosa* at four stimulation frequencies. (*a*) Wavelength of travelling waves in the anterior tympanum (ATM). (*b*) Wavelength of travelling waves in the posterior tympanum (PTM). (*c*) Travelling wave velocity in the ATM. (*d*) Travelling wave velocity in the PTM. Points represent means, error bars indicate ± s.d.

Observations of the displacement and time domain data demonstrated that at high frequencies, the tympana display travelling waves. To characterize the mechanical properties of the travelling waves, wavelength and wave velocity were calculated using existing protocols [9,38]. For the ATM, there was strong evidence that the wavelength of the travelling wave was negatively associated with stimulus frequency, decreasing from 1.2 ± 0.05 mm at 12.5 kHz to 0.84 ± 0.06 mm at 25 kHz (figure 4*a*; ANOVA, *f* = 28.12, *p* < 0.001). The PTM wavelengths displayed a steeper negative trend over this frequency range, with a smaller wavelength at higher frequencies, from 1.3 ± 0.049 mm at 12.5 kHz to 0.43 ± 0.09 mm at 25 kHz (figure 4*b*; ANOVA, *f* = 7.64, *p* < 0.01). For the ATM, there was strong evidence that wave velocity was positively associated with stimulus frequency, from 15.04 ± 0.59 m s^−1^ at 12.5 kHz to 20.94 ± 1.49 m s^−1^ at 25 kHz (figure 4*c*; ANOVA, *f* = 20.3, *p* < 0.001), but for the PTM, there was a strong negative association between wave velocity with increasing stimulus frequency from 16.22 ± 0.62 m s^−1^ at 12.5 kHz to 10.69 ± 2.34 m s^−1^ at 25 kHz (figure 4*d*; ANOVA, *f* = 70.33, *p* < 0.001).

## 4. Discussion

We report that resonances in the tympanal membranes contribute to frequency discrimination in *C. monstrosa*. This mechanism is likely responsible for activation of the appropriate receptor group in their CA by local vibrations of the anterior tracheal wall, above which the CA lies. Both tympana show their greatest average response (combined across all scan points) around 2 kHz, as suggested by existing studies [26,28], and drop in both displacement and velocity towards 30 kHz (figure 2). While at the level of the whole tympanum, there is a mismatch between calling song frequency and the best frequency threshold [28], this mismatch appears to be partially resolved when we consider the frequency and place specific responses of the tympanum, whereby stimulus frequency is divided into two main regions of the tympanum, with a division point at 8.12 ± 0.47 kHz (figure 2). Furthermore, at frequencies above this division, the displacement occurs as a travelling wave, and its response in the distal region of the tympanum becomes tonotopic. The matched filter hypothesis [41,42] would here suggest that afferent nerve activity and the need for high frequency tuning of the CA is reduced, as frequency information is mechanically filtered into two frequency-specific channels prior to central processing [43]. This may initially indicate that the ear of *C. monstrosa* is similar to the two-channel hearing of the gryllid *Teleogryllus oceanicus*, in which the activation of one of two different groups of afferent interneurons occurs, depending on whether the frequency of the stimulus is above, or below 15 kHz [44]. However, unlike *T. oceanicus*, *C. monstrosa* has been found to have higher levels of frequency discrimination at the interneuron level [45]. Thus, the definition of this ear as a two-channel receiver does not extend beyond the mechanical displacement modes of the tympana. These two modalities may therefore simply aid in the tonotopy of the CA, which then begins to further filter vibrations by their frequency composition. Non-invasive mechanical measurements of the CA of *C. monstrosa* by novel methods such as optical coherence tomography [18,46] would be useful in clarifying the relative contribution of the tympana to overall frequency discrimination.

The discrimination of frequency information by distinct resonant modalities is not unique to *C. monstrosa* but also observed in the locust (Caelifera) tympanum, whereby higher frequencies induce higher vibrational modes, permitting location-dependant vibrations of the tympanum [8,9]. Given that the tympana of *C. monstrosa* also appear to follow the expected resonant modes of an ovular membrane [47], we may infer that the evolution of multiple frequency processing organs in tympanal insects has been facilitated by adoption of natural vibrational modes. The same appears to be true of mole cricket (Gryllotalpidae) tympana, which display region-specific reception of different stimulus frequencies in a manner very similar to *C. monstrosa* [8]. However, at the interneural level, like in *C. monstrosa*, there is evidence supporting greater frequency resolution of these ears [48]. The gryllotalpids and the hagloids both evolved in the Jurassic [32,49], and possess relatively simple forms of the foretibial hearing organ, suggesting that some degree of tympanal frequency processing may represent the relict condition from which modern ensiferan ears have evolved. However, given the 100 Myr since the separation of these groups, the similarities in tympana function may have convergently evolved [33]. Either way, the tympana of *C. monstrosa*, with their overall low frequency sensitivity, support hypotheses that the precursor organs to the ensiferan tympanal ear functioned as detectors of substrate-borne and low frequency vibration [28,50], likely for the enhanced detection of terrestrial predators [28,34].

### (a) The evolution of the tettigoniid *crista acustica*

Further insights into the evolution of the ensiferan ear arise from these findings. The division of high and low frequencies into the distal and proximal ends of the tympana and CA offers a functional intermediate between the relict CA homologue which functions for vibration detection [47,50,51] and the complex tettigoniid CA, which is capable of sophisticated tonotopic frequency analysis [10,11,19,48]. In the foretibial organ of *C. monstrosa*, the tracheal branches are symmetrical, and ovular in cross section (figure 1). The CA lies above the anterior tracheal branch, as with modern tettigoniids (figure 1; [14]). The ear of *C. monstrosa* must therefore exploit tonotopic resonances of the tympanum to localize vibrations to different points along the anterior tracheal branch, which in turn activates the nearest mechanoreceptors, which are not directly connected to the tympana. However, due to the fact, the travelling waves are backed by air rather than a fluid (as in the tettigoniids), there should be considerable transmission loss between the external displacement and the CA, particularly for higher sound frequencies. This idea is supported by the neurophysiological recordings of Mason [26,28], whereby the calling song frequency requires increased stimulation to pass the neural threshold.

The observation that the region of the tympanum that responds well to the calling song frequency is physically distal may also provide an explanation for the mismatch in the hearing thresholds of this species [28], as the CA is located towards the proximal end of the hearing organ. Thus more energy would be required to convert this small area of tympanal displacement into a neuronal response as the tympanum displacement is further from the mechanosensory units. High auditory thresholds around the calling song frequency may not be such a problem for conspecific communication in this species, as the male calling song is extremely loud (over 100 dB re. 20 µPa at 20 cm; electronic supplementary material, figure S1). The spatial disparity between the CA and the distal tympanal displacement within the ear could be reduced over time if high ear sensitivity was selected for, because reducing the distance between the mechanoreceptors and peripheral displacement would result in reduced attenuation of the signal throughout the ear for greater mechanical displacement of the chordotonal organ. Modifications to the shape of the anterior tracheal branch could also enhance this reception, and thus we may expect an asymmetric layout of the anterior and posterior tracheal branches to evolve under selection for increased ear sensitivity. This is the case of the foretibial ear of the tettigoniid, whereby the DW of this anterior branch has become wider and flatter [14]. Recently, it was shown in Tettigoniidae that the DW of the anterior tracheal branch is tonotopic and contributes heavily to the mechanotransduction process [18]. Given that the anterior tympanum and mechanosensory units of *C. monstrosa* are mechanically coupled by the anterior tracheal branch like in most Ensiferans [46], we postulate that the tonotopic vibrations of the DW have originated from a tonotopic tympanum, with the vibrations shifting dorsally along the anterior tracheal branch over evolutionary time to reduce internal attenuation of the peripheral mechanical displacement. Observations of the displacement of the tympana of tettigoniid species show that while the CA and DW are tonotopic, the tympana are not [10,11,35], and thus the tonotopic arrangement may have simply shifted to the DW, with the process of hearing involving an additional mechanical step [8,18,35]. Alternatively, the mechanical tonotopy of the tympana of *C. monstrosa* may already be represented in the CA and DW; and in the bushcrickets, enhanced CA and DW tonotopy has simply been favoured over tympana tonotopy, with the latter being lost over evolutionary time.

Existing measurements of the mechanics of the CA in bushcrickets (*Mecopoda* and *Copiphora* spp.) have found several qualities comparable to the tonotopic response of the tympanum of *C. monstrosa* presented here [10,11,38], supporting our evolutionary hypotheses. Most similar is the arrangement of the travelling wave from distal to proximal end across the inner ear [10,11,38] and the orientation of tonotopy; with higher frequencies being received more distally [21]. It has also been found that higher frequency sounds display a greater velocity of travelling wave in the CA [38] and a narrower micromechanical response in the CA and DW [18]. The same observations are made here within the ATM (figures 2 and 4). Furthermore, the vibrations of the DW display a unilateral widening of their mechanical response towards the proximal end of the ear [18]. The same is true of the response of the tympana of *C. monstrosa*. It is concluded therefore, that this mode of frequency analysis is likely to be the mechanical precursor to the tonotopy of the DW of the anterior tracheal branch observed in the tettigoniid ear. The PTM travelling waves on the other hand appear to resemble those of the tympanum of the locust, with a decreasing wavelength and decreasing wave velocity [9]. The role of the posterior tympanum in *C. monstrosa* thus appears to be a broadband receiver, perhaps to similarly enhance vibrations along the entire length of the CA regardless of stimulation frequency. Further comparisons of the similarities between the travelling waves presented here with those of mammalian and invertebrate ears are shown in electronic supplementary material, figure S2.

## 5. Conclusion

*Cyphoderris monstrosa* is a relict species among the Ensifera, sharing an ancient common ancestor with the tettigoniids [33,49]. The finding that the tympanal travelling waves and tonotopy resemble that of the tettigoniid CA evokes the following hypothesis of the mechanism by which the complex ear of the tettigoniids has evolved: natural resonant modalities of the tympanum form a simple tonotopic arrangement for high frequencies, which has been mechanically coupled to the DW over time to reduce the distance between tympanum displacement and the mechanosensory units, increasing hearing sensitivity. Later, the DW and CA have become more specialized for tonotopic reception, convergently evolving similar mechanics to the mammalian cochlea for enhanced frequency resolution [10,11,20]. Comparative investigations of ensiferan tympanal mechanics in a phylogenetic context could prove beneficial in refining this hypothesis.

## Data Availability

Further data, including movies of the raw data during collection, are available in the electronic supplementary material [52].
